# Is a bird in the hand worth two in the future? Intertemporal choice, attachment and theory of mind in school-aged children

**DOI:** 10.3389/fpsyg.2014.00483

**Published:** 2014-05-23

**Authors:** Antonella Marchetti, Ilaria Castelli, Laura Sanvito, Davide Massaro

**Affiliations:** Research Unit on Theory of Mind, Department of Psychology, Università Cattolica del Sacro CuoreMilan, Italy

**Keywords:** intertemporal choice, theory of mind, attachment, school-age children, development

## Abstract

Intertemporal choice is a decision-making dilemma related to outcomes of different entity located at different time points. Economic and psychological literature on this topic showed the phenomen of temporal discounting, i.e., the proclivity to devalue the outcome distant in time on the basis of the time delay necessary to obtain it. The goals of this research are to investigate two different components of intertemporal choice separately, namely time and outcome, in school-age children, and the possible link among such components and the security of attachment style and theory of mind. Ninety one children aged between 6 and 10 years performed two intertemporal choice tasks, first and second order false belief tasks and the Separation Anxiety Task in the Family and School versions. Results showed that the two components of intertemporal choice (waiting tolerance and sensitivity to delayed outcome) are stately interrelated; the quality of the attachment to the family caregiver affects the tolerance to waiting time and first order false belief understanding affects both the components of intertemporal choice.

## Introduction

In everyday life people often face the choice to forgo an immediate benefit to get a bigger benefit later. This phenomenon has been named intertemporal choice, i.e., a decision-making dilemma related to benefits or outcomes of different entity located at different time points (Berns et al., 2007; Paglieri and Castelfranchi, 2008; Marchetti et al., 2009a,b).

The strategies and the behaviors that people adopt to solve such type of dilemma constitute a topic of interest both for economists and for psychologists, because they show how the rational and wise decisions that individuals take about goods of different nature (health, life habits, money…) fail to withstand the challenge of time. The interplay between the magnitude of the outcome, the time delay necessary to obtain the bigger outcome, and the subjective perception of these two elements make intertemporal choice a quite complex decision-making process, far away from the model of the rational *homo oeconomicus* proposed by classical economic theories. Within this framework, decision making tends to reach the maximum possible profit, and the time discount rate is supposed to be constant.

However, evidences showed that people do not always maximize their profits and they often prefer the minor outcome soon available, or they decide for the grater outcome available later, but then they change their mind in the meanwhile, and that the nature of the time discount rate is hyperbolic or quasi-hyperbolic (Ainslie, 1992, 2001). Moreover, the temporal discount rate does not remain the same for any type of good and for any time interval (see Frederick et al., 2002).

A key concept to understand the reasons of the inconsistency of people's behavior is temporal discounting. The preference for an outcome close or distant in time is calculated according to the value of the single outcome (1 euro vs. 10 euro) for its delay (a day vs. 1 month). The value of the outcome soon available makes people shortsighted about the greater benefits they could reach if only they were able to delay gratification and to decide for the bigger outcome in the future: in this sense, their decisions become irrationally myopic (Prelec and Loewenstein, 1997; Ebert and Prelec, 2007). Such lack of foresight can be explained considering intertemporal choice as a complex psychological process, that implies various components (see Berns et al., 2007; Kalenscher and Pennartz, 2008), among which the capacity to delay gratification (Ainslie, 1975, 1992). This component has relevant implications in developmental and educational psychology, because the capacity to wait in order to satisfy one's need or desire and to postpone gratification in order to get a higher reward constitutes a continuous acquisition across infancy and childhood until adulthood and elderly (Green et al., 1994; Read and Read, 2004). Not surprisingly, the construct of the “delay of gratification” has become the way intertemporal choice is studied in developmental psychology. Two experimental procedures are usually employed: the choice paradigm or “delay choice” (Mischel and Metzner, 1962) and the waiting paradigm or “delay maintenance” (Mischel, 1974). In the former the participant has to choose between a smaller but immediate outcome (for example, to receive one candy immediately) and a bigger outcome but delayed over time (for example, three candies next week). In the latter, the procedure is similar, but the participant can decide at any time to stop waiting and to get the smaller but immediate outcome.

Various researches conducted with these procedures (see for example Mischel and Metzner, 1962; Schwarz et al., 1983; Mischel et al., 1989; Shoda et al., 1990; Thompson et al., 1997; Lemmon and Moore, 2001, 2007) mainly focused on the preschool age, showing a significant effect of age on the delay of gratification. Other studies considered the school-age only in a longitudinal perspective, analyzing the predictivity of the delay of gratification in preschoolers in various domains, such as cognition and attention (Mischel and Metzner, 1962; Eigsti et al., 2006), the ability to resist to temptations in general (Mischel and Gilligan, 1964), the assumption of responsibility (Stumphauzer, 1972), the adoption of empathic and prosocial behavior (Krueger et al., 1996; Thompson et al., 1997), the adaptation to the school context (McIntyre et al., 2006) and academic achievement (Bembenutty and Karabenick, 2004; Yang and Wang, 2007), the adoption of successful coping strategies in adolescence and in adulthood (Mischel et al., 1988; Shoda et al., 1990; Ayduk et al., 2000). The pioneer work by Mischel and Metzner (1962) is the only one that investigated the age range from 5 to 12 with a cross-sectional design.

The ability to delay gratification resulted to be sensitive also to factors such as gender—females would be more inclined to postpone gratification—and the degree of emotional involvement of the mother in the relationship with their child, measured by the expression of maternal emotions (Jacobsen, 1998). Moreover, some authors have taken into account the possible links between the ability to delay gratification and the development of social and cognitive abilities, such as the tendency to be prosocial and altruistic (Thompson et al., 1997; Moore, 2009) and theory of mind (Moore et al., 1998; Moore and Symons, 2005).

As for social skills, Thompson et al. (1997) and Moore (2009) proposed a joint study of the ability to delay gratification and other-oriented preference. They presented preschoolers with a number of two alternative choices, contrasting an immediate reward of stickers with four alternatives. The first choice between one sticker for self now or one each for self and partner was a simple measure of the tendency to share. The second choice, instead, examined sharing when there was a cost to self, because the choice was between two stickers for self now or one each for self and partner now. The two remaining choices introduced the time component. The shared delay was a choice between one sticker for self now or one each for self and partner later: in this case, the subject did not sacrifice anything, but he/she had to forego immediate self-gratification in order that the other might benefit. The final choice was a classical delay of gratification, in which the child had to choose between one sticker for self now or two stickers for self later. Results show that the ability to delay gratification develops parallel to the ability to make altruistic choices, and in particular the fourth year of age constitutes a turning point in such a development. This result is very interesting, because the fourth year of age constitutes a cornerstone also in the development of theory of mind. Theory of mind is the ability to interpret and predict our own and others' behavior in terms of mental states (Premack and Woodruff, 1978). It is not an “independent” competence, instead it is one of the several cognitive skills employed in social interactions (Astington, 2003; German and Hehman, 2006; Massaro and Castelli, 2009; Massaro et al., 2013a,b, 2014). Initially, theory of mind research focused on childhood, identifying the steps of its development, mainly using false belief tasks. More recently, researchers adopted a life span perspective, considering adults and the elderly (Henry et al., 2006; Castelli et al., 2010). Results showed that theory of mind is not an “all or nothing” competence, rather it continues to undergo changes across life. More specifically, according to Apperly (2012) the transition from childhood to adulthood may be characterized by an increase in theory of mind flexibility and speed of usage. Furthermore, the pattern of correlation of theory of mind with other socio-cognitive abilities changes across development. In the light of these recent evidences theory of mind development could be intended as spiral pathway, where the aspects of competence and awareness increase cyclically.

To the best of our knowledge, there are few works that have studied the possible link between intertemporal choice and theory of mind, namely the works by Moore et al. (1998) and Moore and Symons (2005). The first work tested theory of mind through classical false belief tasks and evaluated the ability to delay gratification with same set of choices of the work by Thompson et al. (1997). Results showed that in 4-years-old children good performances in false belief tasks were significantly correlated with the tendency to delay to share in the choice between one sticker now or one each later, and close to significance with the tendency to delay to share in the choice between one sticker self now or one each later. Such findings suggest that a good level of theory of mind supports the child's ability to take the perspective of the other person and also to manage it in the future, thus making an altruistic choice and delaying gratification too. Moore and Symons (2005) continued on this path of research, adding also a new construct, i.e., the quality of attachment. In fact, the ability to delay gratification is connected to the ability of self-regulation, which in turn is strongly related to the pattern of attachment.

According to the theory of attachment (Bowlby, 1969, 1973, 1980), humans are characterized by a universal need to create and maintain a deep emotional relationship with their caregivers. The aim of the attachment system is the experimentation of security, which in turn acts as a regulator of the emotional experience. The most famous research paradigm devised to evaluate attachment is the Strange Situation (Ainsworth et al., 1971) that has allowed the identification of three styles of attachment, i.e., secure, insecure-avoidant, insecure-ambivalent, followed by the disorganized style identified later by Main and Solomon (1986). The type of attachment is crucial for the subsequent child development, because on the basis of the quality of the relationship with the caregiver the child elaborates an “Internal Working Model” (Bowlby, 1973), i.e., a cognitive representational structure which is linked to the conception of the Self as worthy of receiving care, basis of self-esteem, and to the conception of one's own self-efficacy, basis of self-confidence. The latter is rooted in the trustful relationship that children develop with the primary caregiver in the early years of life.

Jacobsen et al. (1997) were the first to investigate the possible link between delay of gratification and attachment style. They found that the quality of the attachment relationship assessed at 12 and 18 months of age with the Strange Situation is predictive of the ability to deal with the classical delay of gratification task at 6 years of age, with an average waiting time higher for children with a secure attachment compared to children with insecure attachment. This result confirms the evidence, already present in the literature, about the difficulty in self-regulation and in the control of impulsivity for the insecure attached children (see for example, Lyons-Ruth et al., 1993; Shaw and Vondra, 1995).

Moore and Symons (2005) explored the relationship between the quality of the attachment relationship at three and a half years of age and various competences at 4 years of age: theory of mind (measured as false belief understanding), prosociality and intertemporal choice (measured with two modified versions of the delay choice paradigm: two stickers for you now vs. two stickers to be shared now; two stickers for you now vs. two stickers to be shared in the future), and executive control (measured by the gift delay paradigm, where the child can receive a gift only if she/he turns to the wall while the gift is being wrapped). They found that secure attachment, in addition to being linked to theory of mind (Fonagy et al., 1997; Meins et al., 2002; Liverta Sempio et al., 2005), is related to the intertemporal choice and to executive control. No links between attachment and prosociality were highlighted.

On the basis of this overview, the goal of our research is to study the possible link among attachment style, theory of mind and intertemporal choice in primary school age children with a cross-sectional design. Our work has two innovative aspects. The first one is the choice of the age period, because as explained before the literature about the delay of gratification has focused mainly on the preschool period. The study of the development of the ability to delay gratification in primary school-age children is interesting because during this period formal instruction requires pupils to improve in their ability to remain focused on cognitive tasks, avoiding distractions and postponing gratification of other goals. Therefore, considering 6, 8, and 10 year olds can allow to observe the possible changes in the delay of gratification over the entire primary school period. The second one is the focus on the two core components of intertemporal choice separately, namely the increase in waiting time and the increase in the magnitude of the outcome. In fact, in the classical choice paradigm used to measure intertemporal choice these two components are entangled, because the child or the adult is required to make a choice between a small outcome immediately or a bigger outcome later. In this way, no information about the subject's sensitivity to the waiting time and to the magnitude of the outcome separately is provided. The same happens for the other methods used to assess intertemporal choice. In children the delay maintenance task provides a measure of the persistence in the decision, and in adults the classical methods again do not address such two components separately: in the matching tasks respondents “fill in the blank” to equate two intertemporal options (100 euro now = … in 1 year), in the rating tasks subjects evaluate an outcome occurring at a particular time by rating its attractiveness or aversiveness, and in the pricing tasks subjects specify a willingness to pay to obtain (or avoid) some real or hypothetical outcome occurring at a particular time (Frederick et al., 2002).

We think that focusing on the two core components of intertemporal choice separately may be useful to further understand their specific contribution and the strict interplay between them. In fact, literature about the neural basis of intertemporal choice has provided strong evidence of the fact that those components are processed by specific neural circuits (for a review see Kalenscher and Pennartz, 2008), although a crucial topic of debate regards the neural site for the integration of the two separate neural systems. Roesch and Olson (2005) trained monkeys in two tasks, one with a variable delay and a fixed reward amount, and another one with a variable reward, but fixed delay. They found that reward proximity and quantity are processed by the same orbitofrontal neurons, implying they may be integrated on a single-cell level. So, the orbitofrontalcortex (OFC) seems to be the best candidate for such integration at very deep levels of neuroanatomical analyses also in humans. The task devised by Roesch and Olson (Roesch and Olson, 2005) inspired the task we created for our research.

## Aims and hypotheses

The goals of our research are to investigate the two above-described core components of intertemporal choice separately, i.e., the increase in waiting time and the increase in the magnitude of the outcome, and to study them in the primary school-age. We also aim to explore the possible link between the increase in waiting time and the increase in the magnitude of the outcome and attachment style, as well as the possible link between the increase in waiting time and the increase in the magnitude of the outcome and theory of mind.

We hypothesize that the ability to delay gratification, measured through the increase in waiting time and the increase in the magnitude of the outcome, would improve with age because of the development of the capacity to inhibit immediate gratification and to tolerate the frustration. As regards theory of mind, measured as the capacity to understand others' mental states to predict the behavior (1st and 2nd order false belief tasks), we hypothesize that it may support children with both of the measures of intertemporal choice. In fact, the development of meta-representational abilities may help to prefigure and manage the representation of a postponed advantage and drive the children to delay gratification. For this reason this interaction may depend on age. Conversely, we hypothesize that theory of mind, measured as a purely metalinguistic ability (metacognitive vocabulary), would not predict the two measures of the ability to delay gratification because not directly rooted into the meta-representational mechanism. Finally, we hypothesize that attachment style to the family and the school caregiver would predict the ability to delay gratification, measured through the increase in waiting time but not to the increase in the magnitude of the outcome, because only the former is closely linked to the development of trust in other people.

## Method

### Participants

Ninety-one children took part in the study. The attended a public Primary school in Northern Italy. After the acknowledgement of the Director of the school to carry on the research, families were contacted through the teachers and were asked to provide informed written consent for their children to be enrolled in the study. No one refused to give the consent. Children were divided as follows: the first group, classified as 6-year olds (*N* = 30, 18 boys, 12 girls, *M* = 6.4 years, *SD* = 0.31); the second group classified as 8-year-olds (*N* = 29, 17 boys, 12 girls, *M* = 8.3 years, *SD* = 0.31); the last group, classified as 10-year-olds (*N* = 32, 20 boys, 12 girls, *M* = 10.4 years, *SD* = 0.26). Participants belonged to the middle socio-economic status based on the parents' education and socio-economic level. Children were neither referred to social services nor reported by teachers for learning and socio-relational difficulties.

### Procedure

Children were submitted to the following battery of tasks. The intertemporal choice task, appositely devised for the present research, consists of two procedures: procedure A, based on time variation, and procedure B, based on outcome variation. Theory of mind was evaluated with three tasks: a classical 1st order false belief task (the unexpected transfer task: Wimmer and Perner, 1983), a classical 2nd order false belief task (the ice-cream story: Perner and Wimmer, 1985), and the metacognitive vocabulary (Astington and Pelletier, 1998). The attachment style was investigated with the Separation Anxiety Test (SAT) in the family version and in the school version (Liverta Sempio et al., 2001). Finally, the Peabody Picture Vocabulary Test-Revised (PPVT-R) was used as a language control task (Dunn and Dunn, 1981; Stella et al., 2000).

All the tasks were submitted in a fixed order, following the above list. Each child was tested individually in a quiet room at school, and required about 40–45 min to complete the test session.

### Tasks

#### The intertemporal choice task (IC)

This task was appositely devised for the present research. It has two innovative aspects. First, it does not offer a single choice between one outcome now and two or more outcomes later, but it proposes a continuous set of choices. Second, it assesses the ability to manage the two components of intertemporal choice separately, i.e., the entity of the waiting time (procedure A) and the entity of the outcome (procedure B).

***Procedure A.*** Children were explained that the aim of the first task was to know how many days, starting from 7 days and decreasing until 2 days, they would have tolerated to wait in order to get two candies instead of one candy immediately. So, the set of choices consisted in a fixed amount of candies (one candy now vs. two candies later) with a decreasing waiting time (7, 6, 5 days, and so on). The set of choices started in this way: “Today is Tuesday: I can give you one candy now or two candies within 7 days, i.e., next Tuesday. What do you prefer?” then “Today is Tuesday: I can give you one candy now or two candies within 6 days, i.e., next Monday. What do you prefer?” and so on. The task is stopped once the child decides for the greater outcome later, i.e., two candies in the future. The score is given to the set of choices on a seven point scale, where one score is given to the absence of temporal delay, i.e., the child chooses one candy immediately, and seven is given to the highest temporal delay, i.e., the child chooses two candies within 7 days.

***Procedure B.*** Children were explained that the aim of the this task was to know how many candies, starting from two and increasing until seven, they would have considered good enough to wait for one week to get them. The set of choices consisted in an increasing amount of candies later on (one candy now vs. two/three/four/five/six/seven candies in 1 week) within a fixed waiting time (one week). The set of choices started in this way: “Today is Tuesday: I can give you one candy now or two candies within 7 days, i.e., next Tuesday. What do you prefer?” then “Today is Tuesday: I can give you one candy now or three candies within 7 days, i.e., next Tuesday. What do you prefer?” and so on. The task is stopped once the child decides the sufficient number of candies to wait for one week. The score is given to the set of choices on a seven point scale, where one score is given to the highest number of candies (seven) within one week, and seven to the lowest number of candies (two) within one week.

The transcription of the answers was performed by two independent coders. Any discrepancy was discussed and solved collegially. Two different scores are obtained: the score of the procedure A is an indicator of the child's waiting tolerance to get a bigger outcome, whereas the score of procedure B is an indicator of the child's sensitivity to delayed outcome amount.

Children received the amount of candies related to their choices in each procedure.

#### Theory of mind tasks

A task exploring the ability to manage verbs referring to mental activities was employed. A first-order and a second-order false belief tasks supported by illustrations were used to examine children's ability to perform first and second-order levels of recursive thinking, namely “I think that you think that…” and “I think that you think that he/she thinks that….” These tasks were chosen because considered by the literature sufficiently reliable and valid in measuring the constructs explored (Wellman et al., 2001).

#### Metacognitive Vocabulary Test (METVOC)

The Metacognitive Vocabulary test (Astington and Pelletier, 1998) was used to measure children's competences about mentalistic verbs that express their own and other people's mental states, such as “to understand, to forget, to know” and so on. This task is based on 12 short stories accompanied by pictures. Children have to choose which is the best verb that expresses the mental state of the character, choosing in a set of two alternatives. For example: *“Dad comes into the room and says: “Time for bed. If it's sunny tomorrow, we'll go to the park.” In the morning John gets out of the bed and looks out the window: he sees the rain pouring down. “Oh no” says John, “Look at that! We won't be going to the park today.”* After the story, the child is asked if John “knows” that it is raining or if he “remembers” that it is raining. The right answer is that John “knows.”

Correct answers were coded with 1 point, whereas wrong answers with 0 point. Children can obtain a total score that ranges from 0 to 12 points.

***First-order false belief task (1st order FB).*** It is a classical unexpected transfer task (Wimmer and Perner, 1983). The story is about Antonio and Francesco, which are playing with a ball in a bedroom. The doorbell rings, so Antonio puts the ball in a wardrobe and goes to the door. In the meanwhile, Francesco takes the ball out of the wardrobe and puts it under the bed. The story ends when Antonio comes back to the room and wants to play with his ball. At this point the child is asked the following questions: “Where will Antonio look for the ball?” (First-order false belief question), “Why?” (Justification question) and two control questions about memory and reality.

The answers to the two control questions about memory and reality were used to filter the children's performance: children who did not pass them were scored as 0. The test question about the false belief was scored as 1 if correct and 0 if incorrect. The justification question was scored 0 if incorrect, 1 if correct but without references to the mental activity, 2 if correct and with references to the mental activity.

***Second-order false belief task (2nd order FB).*** It is a classical unexpected transfer task (Perner and Wimmer, 1985). The story is about Maria and Giovanna, which are playing in the park when they see an ice-cream van. Maria wants to get an ice-cream, but she has no money, so she decides to go home in order to take the money, being sure that the ice-cream van will remain in the park. But while Maria is away, Giovanna sees the ice-cream van moving away and asks to the ice-cream man where he is going. He says that he is going in front of the school to sell more ice-creams. While Maria is at home, she sees the ice-cream man and asks him where he is going, so that she comes to know that he is moving outside the school. At the end of the story Giovanna goes to Maria's house and asks her mother where her friend is: Maria's mum answers that Maria is just gone out to buy an ice-cream. At this point the child is asked the following questions: “Where does Giovanna think that Maria thinks the ice-cream van is?” (second-order false belief question), “Why?” (Justification question), and two control questions about memory and reality. The scoring was the same as described for the first-order false belief task.

#### Separation anxiety test–family version and school version (F-SAT and S-SAT)

The Separation Anxiety Test is a semi-projective task that evaluates the child's mental representation of the attachment style with the caregiver. The original version was elaborated by Hansburg (1972) for adolescents between 11 and 17 years, then Klagsbrun and Bowlby (1976) have adapted this version for children from 4 to 7 years. Their new task consisted of six pictures, each one representing a situation with a separation from the familiar caregiver: three situations deal with strong intensity separations, and three with medium intensity separations. The child is asked to describe the protagonist's feelings, to justify them, and to foresee what the protagonist will do, i.e., the coping strategy of the protagonist. The Italian version by Liverta Sempio et al. (2001) was used for this study. This version is the result of an adjustment from the other versions of the same task (Fonagy et al., 1997; Slough et al., 1988) and has the advantage of allowing the evaluation of the quality of the attachment style not only with the family caregiver, but also with the school caregiver.

The coding system is the one provided by Liverta Sempio et al. (2001), based on the previous one defined by Slough and Greenberg (1990) and by Fonagy et al. (1997). This system is organized on three dimensions: attachment, i.e., the ability to express vulnerability and need (measured through the three situations of high intensity of separation); self-confidence, i.e., the ability to face separation in an autonomous mood (measured through the three situations of medium intensity of separation) and avoidance, i.e., the propensity to speak about the separation (measured through all the six situations of separation). Each participant received one score for each dimension. More specifically, the attachment and self-confidence scales have a score ranging from 3 to 12 (range 1-4 for each of three situations), while the avoidance scale has a score ranging from 6 to 18 (range 1-3 for each of the six situations). Then the total attachment score was calculated. This final score is the result of the sum of the scores in the attachment scale and in the self-confidence scale, and of the sum of the inverse of the avoidance scale, calculated by subtracting this score from the total amount potentially obtainable in this scale. We decided to use this task because in the literature it has been successfully employed for the assessment of attachment style in conjunction with the assessment of theory of mind in samples of preadolescents (Fonagy et al., 1997; Lecciso et al., 2011).

#### The peabody picture vocabulary test-revised

The Peabody Picture Vocabulary Test—Revised (PPVT—R) is a standardized test used to measure the receptive vocabulary and it is useful in order to reveal high or low verbal abilities (Dunn and Dunn, 1981; Stella et al., 2000).

This test is constituted of 175 items, organized on different levels of difficulty from simple ones to difficult ones. A series of pictures were presented to the participant, since each page included four pictures. The experimenter stated a word, describing one of the pictures, and asked the child to point to or to say the number of the picture that the word described. Each child was not presented with all the items but only with the ones that were in the critical interval defined by items on an inferior level, the basal one, and on a superior level, the ceiling one. So for all subjects it was necessary to determine the first item on the basis of the chronological age, then it was defined the basal level and the ceiling one. Standard scores range from 40 to 160.

## Results

The PPVT-R revealed no significant age differences among the three age groups. Therefore, the entire sample satisfied the prerequisite of adequate language understanding in order to be submitted to the battery of tasks [*F*_(2, 88)_ = 2.834 *p* = 0.064].

Table 1 reports the descriptive statistics for the explored variables. First, a preliminary evaluation of the link between the two IC measures as well as the effect of gender and age on them are presented. Thus, the possible links among IC measures, and language variables, theory of mind performance, and attachment scores are explored. Finally, a general model that overall evaluates the impact of gender and age as well as of theory of mind and attachment on the IC scores is presented.

**Table 1:** **Descriptive statistics of the tasks performances**.

**Age group**		***N***	**Minimum**	**Maximum**	**Mean**	**Std. deviation**
6-years	PPVT	30	84	116	103.23	8.557
	METVOC	30	7	11	9.67	1.061
	1st OR. FB	30	0.00	2.00	1.5333	0.81931
	2nd OR. FB	30	0.00	3.00	0.6000	0.96847
	F-SAT	30	11	34	27.70	4.728
	S-SAT	30	17	35	28.87	4.345
	A-IC	30	0	5	3.23	1.633
	B-IC	30	0	5	2.77	2
8-years	PPVT	29	74	125	109.14	8.963
	METVOC	29	9	12	10.83	0.928
	1st OR. FB	29	2.00	3.00	2.1034	0.30993
	2nd OR. FB	29	0.00	3.00	0.8276	1.03748
	F-SAT	29	20	36	29.72	3.788
	S-SAT	29	16	36	29.17	4.706
	A-IC	29	0	5	4.10	1.589
	B-IC	29	0	5	3.86	1.642
10-years	PPVT	32	75	130	108.63	13.445
	METVOC	32	10	12	11.53	0.671
	1st OR. FB	32	2.00	3.00	2.3125	0.47093
	2nd OR. FB	32	0.00	3.00	1.2500	1.24434
	F-SAT	32	21	34	30.66	3.798
	S-SAT	32	22	34	30.62	2.768
	A-IC	32	0	5	4.38	1.238
	B-IC	32	0	5	4.66	1.004

The two IC scores were strongly and high significantly correlated (*r* = 0.753, *p* < 0.001). The more children were able to wait longer in order to obtain two candies instead of one immediately, the more they were sensitive to a small increase of the outcome in order to wait instead of choosing one candy immediately. The *T*-test for paired samples did not show any significant difference between these two performances (*p* = 0.29). Anovas showed that Gender did not influence the IC performances (*p* > 0.15), whereas an age effect on both A- and B- IC scores was revealed [respectively, *F*_(2, 88)_ = 4.892, *p* < 0.05; *F*_(2, 88)_ = 10.817, *p* < 0.001]. In both cases, the 10 year olds wait significantly more than children aged 6 (respectively, *p* < 0.05 and *p* < 0.001).

The bivariate correlations highlighted several positive and significant links among IC scores and the other variables explored (see Table 2).

**Table 2:** **Correlations among intertemporal choice measures and the other study variables**.

	**F-SAT**	**S-SAT**	**METVOC**	**1st order FB**	**2nd order FB**
IC-A	0.387^**^	0.255^*^	330^**^	0.390^**^	0.072
IC-B	0.251^*^	0.243^*^	0.425^**^	0.494^**^	0.090

* p < 0.05 level.

** p < 0.01 level.

Both the IC-A (waiting tolerance) and the IC-B (sensitivity to delayed outcome amount) were significantly and positively correlated with, F-SAT, S-SAT, METVOC and 1st order FB. Partial correlations controlling for age were performed again revealing that some significant links were independent by developmental processes (see Table 3). IC-A correlated with, F-SAT, S-SAT and with 1st order FB. The IC-B correlated with 1st order FB. Given the absence of correlations between the measures of intertemporal choice and 2nd order false belief understanding, the latter was excluded from subsequent analyzes.

**Table 3:** **Partial correlations among intertemporal choice measures and the other study variables controlling for age**.

	**F-SAT**	**S-SAT**	**METVOC**	**1st order FB**	**2nd order FB**
IC-A	0.323^**^	0.210^*^	0.178	0.283^**^	−0.009
IC-B	0.131	0.179	0.188	0.340^**^	−0.032

* p < 0.05 level.

** p < 0.01 level.

On the basis of these preliminary results we run a Univariate General Linear Model in order to explore the effect of age on the intertemporal choice performance obtained by summing the two IC scores, controlling for F-Sat, S-SAT METVOC, 1st FB understanding, Although the unique contribute of age was highly significant, accounting for the 14.8% of the total variance of the intertemporal choice performance [*F*_(2, 88)_ = 8.816, *p* < 0.001, θ = 0.967], the covariates share enough variance in common with intertemporal choice, cancelling the main effect of age. In particular, only the first-order false belief understanding seems to explain a significant part of the variance of the intertemporal choice, net of other covariates [*F*_(2, 82)_ = 6.512, *p* < 0.05, η*p*^2^ = 0.074, θ = 0.713].

To examine the unique contribution of age, attachment and theory of mind in the explanation of the IC-A and IC-B, a three steps hierarchical multiple regression analysis was performed. In particular, step 1 introduces the age in the model, step 2 the measures of attachment (F-SAT and S-SAT) and step 3 the measures of theory of mind (1st order FB and METVOC). With regard to IC-A measure, in all three models a significant change in R^2^ was found (see Table 4).

**Table 4:** **Model and R^2^ Change summary predicting IC-A**.

**Model**	***R***	***R* square**	**Adjusted *R* square**	**Std. error of the estimate**	**Change statistics**				
					***R* square change**	***F* change**	***df*1**	***df*2**	**Sig. *F* change**
1	0.310^a^	0.096	0.086	1.486	0.096	9.461	1	89	0.003
2	0.447^b^	0.200	0.172	1.414	0.104	5.649	2	87	0.005
3	0.516^c^	0.266	0.223	1.370	0.066	3.827	2	85	0.026

a Predictors: (Constant), Age.

b Predictors: (Constant), Age, F-SAT, S-SAT.

c Predictors: (Constant), Age, F-SAT, S-SAT, METVOC, 1st ORDER FB.

Step 1 indicated that age significantly predicted the intertemporal choice [*F*_(1, 89)_ = 9.46, *p* < 0.01, β = 0.31, *t* = 3.08, *p* < 0.01]. In step 2 [*F*_(1, 89)_ = 7.25, *p* < 0.001], although the age continued to be a significant predictor (ß = 0.21, *t* = 2.10, *p* < 0.05), the F-SAT significantly contributed to the explanation of intertemporal choice (ß = 0.32, *t* = 3.20, p < 0.01). Finally, in step 3 [*F*_(1, 89)_ = 6.16, *p* < 0.001], 1st order FB predicted intertemporal choice (ß = 0.27, *t* = 2.41, *p* < 0.05). Even the F-SAT continued to have a significant role (ß = 0.24, *t* = 2.56, *p* < 0.05), while the age ceased to contribute to the explanation of the variance (ß = 0.01, *t* = 0.04, *p* = 0.971). In reference to IC-B, only the model 1 and the model 3 determine a significant change in R^2^ (see Table 5).

**Table 5:** **Model and R^2^ Change summary predicting IC-B**.

**Model**	***R***	***R* square**	**Adjusted *R* square**	**Std. error of the estimate**	**Change statistics**				
					***R* square change**	***F* change**	***df*1**	***df*2**	**Sig. *F* change**
1	0.458^a^	0.210	0.201	1.581	0.210	23.603	1	89	0.000
2	0.489^b^	0.239	0.213	1.569	0.029	1.682	2	87	0.192
3	0.581^c^	0.338	0.299	1.481	0.099	6.338	2	85	0.003

a Predictors: (Constant), Age.

b Predictors: (Constant), Age, F-SAT, S-SAT.

c Predictors: (Constant), Age, F-SAT, S-SAT, METVOC, 1st ORDER FB.

More specifically, in step 1 [*F*_(1, 89)_ = 23.60, *p* < 0.001], the age explained a significant part of the variance of the intertemporal choice (ß = 0.46, *t* = 4.86, *p* < 0.001). In step 2 [*F*_(3, 87)_ = 9.11, *p* < 0.001], the measures of attachment did not contribute to the explanation of variance in a statistically significant manner (F-SAT: ß = 0.08, *t* = 0.69, *p* = 0.49; S-SAT: ß = 0.14, *t* = 1.35, *p* = 0.18), leaving age as the only predictor (ß = 0.41, *t* = 4.16, *p* < 0.001). Finally, in step 3 [*F*_(5, 85)_ = 8.67, *p* < 0.001], the first-order false belief understanding became the only significant predictor of intertemporal choice (ß = 0.32, *t* = 3.07, *p* < 0.01).

## Discussion

This research aimed to investigate intertemporal choice in primary school age children. Intertemporal choice was operationalized as waiting tolerance and sensitivity to delayed outcome amount in order to explore the different single components—namely time and entity of the outcome—which are stately interrelated in this type of decision making dilemma. Furthermore, the effect of age as well as the predictivity of attachment and theory of mind on intertemporal choice measures were evaluated.

The results show that the two components of intertemporal choice are strongly correlated, in the sense that the more children have a high waiting tolerance in order to obtain a little more than what they could obtain immediately, the more they are sensitive to small increase in the entity of the delayed outcome. Conversely, the children who show a low waiting tolerance are those who need a remarkable increase of the amount of the delayed outcome in order to wait for it. This result is interesting at least for two reasons: first, it reveals a behavioral coherence in the intertemporal choice measures, supporting the idea that the coordination of the representations both of the time waiting and of the entity of the outcome is a crucial aspect for this decisional dilemma. Second, this correlation is a new evidence in the literature about intertemporal choice. In fact, the majority of the studies highlights the absence of a link between the two classical ways to assess intertemporal choice (delay choice and delay maintenance) (Schwarz et al., 1983) as also confirmed by a recent review about the convergent validity of multi-method measures of delay tolerance (Duckworth and Kern, 2011). So, our results are in contrast with the main findings of the literature. They help to renew the questions about what the tasks actually measure and how these measurements interface with the constructs theoretically developed. In fact, it is not so clear if the traditional delay choice task and the delay maintenance task actually measure two aspects of the same competence. In the first case, the propensity to make an intertemporal choice is measured; in the second case, the ability to maintain the choice done and so to tolerate waiting is measured. In our work, instead, we measure the tolerance of waiting as well as the cost of such tolerance, adding evidence on children to the result provided on monkeys by Roesch and Olson (Roesch and Olson, 2005). Our result of the strong correlation supports behaviorally the evidence that separate neural activations processing time and amount find a synthesis in the OFC. Furthermore, the two measures of intertemporal choice employed, although correlated, show the differential effects that the other investigated variables (in particular attachment) have on each of them.

We found an effect of age on intertemporal choice coherent with the literature. Interestingly, this effect appears when it is evaluated alone, and it disappears when controlling for other abilities. This is interesting because it shows that, on one hand, the simple increase of age influences the ability of intertemporal choice and, on the other hand, the development of specific cognitive abilities in this period is so significant for the ability under study to neutralize the effect of age.

The quality of the attachment style influences intertemporal choice: this result is interesting because it extends to the school-age period the evidence found in the preschool age by Jacobsen et al. (1997) and Moore and Symons (2005). But our result adds two new evidences. First, attachment influences one specific component of intertemporal choice, namely the tolerance of waiting time. In other words, not only secure children are better able to delay gratification compared to insecure children, but they also tolerate a longer waiting time compared to insecure children. This result is really pertinent, because it confirms the role of attachment on self-control, which is necessary to tolerate waiting, and this is exactly the component of intertemporal choice measured with the procedure A of our task. Second, only attachment style with the family caregiver but not with the school caregiver has this effect, thus confirming the crucial role of the primary caregiving in shaping a trustful relationship that is a prerequisite for self-confidence. However, despite the important role of attachment on the ability to delay gratification, attachment alone does not explain the age variability. So, it surely is a component that deserves to be considered to explain children's ability in intertemporal choice, though not enough to account for the entire process.

Finally, theory of mind has an effect on both components of intertemporal choice, i.e., the tolerance to the waiting time and the sensitivity to the outcome increase. This result is in line with the results of the literature (Moore et al., 1998; Moore and Symons, 2005) and is really interesting given the type of task we have devised to study the two components of intertemporal choice separately. In fact, this result supports the idea that the separate processing of the two components of intertemporal choice arrive at a point of synthesis in the site of the OFC, because OFC is one of the neural sites of theory of mind processing (Shamay-Tsoory et al., 2010).

Theory of mind, measured as the ability to explicit the presence of a recursive thought, makes the effect of age null. We expected that 2nd order false belief reasoning had on primary school-age children the analogous effect that the 1st order false belief reasoning has on preschoolers, because the school-age period is the sensible period for the development and solidification of this more complex level of recursive thinking, but this was not the case. Probably such a level of complex meta-representation is not necessary to manage the task of intertemporal choice, where the participant has to articulate only a single perspective on reality, for which the first order false belief understanding is enough.

Concluding, the intertemporal choice is confirmed as a complex psychological process, which involves various abilities. We have showed the relevance of cognitive as well as emotional dimensions in this process, which deserve future investigations. In fact, one limitation of this study is the study of theory of mind as a purely cognitive ability. So, a promising strategy of future research could be focusing on the different components of theory of mind, adding the analyses of “affective” theory of mind to the analyses of the more cognitive one investigated here. A second limitation is that among the variables belonging to the family context we considered only the attachment style. Other variables in this domain deserve future investigation, for example the educational style of the family for what concerns specifically the habits and rules regarding the delay of gratification. Finally, we employed a cross-sectional design to examine an age range that has been quite neglected so far. It could be interesting to devise longitudinal studies (similar to those on preschoolers described in the introduction) to follow the subsequent development of the delay of gratification and its predictive role on other socio-cognitive competences in adolescence. In fact, we think that this age period is particularly challenging for the evolution of the biological and psychosocial factors underlying intertemporal choice, and so our study could represent a first step in this direction.

### Conflict of interest statement

The authors declare that the research was conducted in the absence of any commercial or financial relationships that could be construed as a potential conflict of interest.
